# Comparison of Different Molecular Weights of Intra-Articular Hyaluronic Acid Injections for Knee Osteoarthritis: A Level I Bayesian Network Meta-Analysis

**DOI:** 10.3390/biomedicines13010175

**Published:** 2025-01-13

**Authors:** Filippo Migliorini, Nicola Maffulli, Cornelis Hindriks Nijboer, Gaetano Pappalardo, Mario Pasurka, Marcel Betsch, Joshua Kubach

**Affiliations:** 1 Department of Orthopaedic and Trauma Surgery, Academic Hospital of Bolzano (SABES-ASDAA), 39100 Bolzano, Italy; migliorinimd@gmail.com; 2 Department of Life Sciences, Health, and Health Professions, Link Campus University, Via del Casale di San Pio V, 00165 Rome, Italy; 3 Faculty of Medicine and Psychology, University La Sapienza, 00185 Roma, Italy; 4 School of Pharmacy and Bioengineering, Keele University Faculty of Medicine, Stoke on Trent ST4 7QB, UK; 5 Centre for Sports and Exercise Medicine, Barts and the London School of Medicine and Dentistry, Mile End Hospital, Queen Mary University of London, London E1 4DG, UK; 6 Department of Surgery, Eifelklinik St. Brigida, 52152 Simmerath, Germany; 7 Department of Orthopaedic Surgery, Oberlinklinik, 14482 Potsdam, Germany; 8 Department of Orthopaedic Surgery, Pineta Grande Hospital, 81030 Castel Volturno, Italy; 9 Department of Trauma and Orthopaedic Surgery, Friedrich-Alexander-University Erlangen-Nuremberg, University Hospital Erlangen, 91054 Erlangen, Germany

**Keywords:** knee, osteoarthritis, hyaluronic acid, injections

## Abstract

**Background:** The present Bayesian network meta-analysis compared the efficacy of intra-articular injections of different molecular weights of hyaluronic acid (HA) in patients with knee osteoarthritis. **Methods:** In November 2024, the following databases were accessed: PubMed, Web of Science, Google Scholar, and Embase. All randomised controlled trials investigating the efficacy of intra-articular HA injections for knee osteoarthritis were accessed. The outcome of interest was to evaluate pain according to the visual analogue scale (VAS). The groups included for comparison were the ultra-high molecular weight (UHMW), high molecular weight (HMW), medium molecular weight (MMW), and low molecular weight (LMW). **Results:** Data from 9822 patients were collected. The mean age of the patients was 62.1 ± 5.0 years with given comparability at baseline. Different follow-up periods were compared. The longest control period ranged from four to six months, and the UHMW and HMW injections were the interventions associated with the greatest reduction in the VAS. LMW HA was the intervention associated with the lowest decrease in VAS, falling short of the control group. **Conclusions:** The main findings of the present Bayesian network meta-analysis, with a current level I of evidence, suggests that the UHMW and HMW HA has a beneficial effect on pain at 6 months post intervention in patients with knee osteoarthritis.

## 1. Introduction

Knee osteoarthritis (OA) is common [1,2]. Knee OA is characterised by progressive cartilage damage with the advancement of bony sclerosis and osteophytes, malalignment, and loss of congruency of the knee joint [3,4,5]. Clinically, patients present with progressive pain and loss of function [6,7,8]. The demographic changes in our society have led to an increase in the prevalence of knee OA, combined with an increasing burden not just for individual patients but also for the healthcare system [2,9]. OA has no cure, but its symptoms can be managed non-operatively or operatively [10,11]. Conservative management includes physical therapy, weight loss, pharmacotherapy, and intra-articular injections [1,11,12,13]. However, inconclusive evidence exists regarding the most effective type of injection, the type of medication used, and the injection regime [7,12,14,15,16,17,18]. 

Intra-articular injection agents are corticosteroids, stromal vascular fraction, platelet-rich plasma, plasma rich in growth factor, bone marrow aspirate concentrate, autologous conditioned serum, and hyaluronic acid [7,12,14,15,16,17,18]. Hyaluronic acid (HA) is a linear glycosaminoglycan composed of D-glucuronic acid and N-acetylglucosamin. Intra-articular HA injections are routinely used in the conservative management of knee OA [13,19,20,21]. HA is an extracellular matrix component found in many tissues [22]. The molecular weight (MW) of HA has a major influence on the rheologic properties in synovial fluid, thus resulting in decreasing joint friction to prevent further cartilage damage [23]. Intra-articular HA is available in a wide range of MWs, with inconsistent nomenclatures of thresholds [13,21]. Low molecular weight (LMW) is defined as 500–1500 kDa; medium molecular weight (MMW), from 1500 to 3000 kDa; high molecular weight (HMW), from 3000 to 6000 kDa; and ultra-high molecular weight (UHMW), more than 6000 kDa [24,25,26]. Different MWs of HA leads to varying anti-oxidative and anti-inflammatory properties [27]. In vitro, LMW and MMW HA induce a pro-inflammatory response, while HMW and UHMW HA result in an anti-inflammatory response with phenotypic changes in macrophages [28]. Furthermore, HMW HA causes an inhibition of Interleukin-6-induced matrix metalloproteinases (MMPs) in human chondrocytes [29]. These molecular changes result in growing evidence that higher-molecular-weight HA facilitates an anti-inflammatory response with the suppression of cartilage degradation. Thus, evidence for the different efficacy of HA products with various MWs has been accumulated [24,27,30,31]. 

Ambiguities exist regarding the optimal molecular weight of HA used for knee OA. No research has been carried out to compare different HA preparations in discrete molecular weight steps. Therefore, the present Bayesian network meta-analysis compared the efficacy of intra-articular injections of four different molecular weight groups in patients with knee OA. The outcome of interest was to evaluate pain according to the visual analogue scale (VAS).

## 2. Methods

### 2.1. Eligibility Criteria

All randomised controlled trials (RCTs) investigating the efficacy of intra-articular HA injections for knee OA were accessed. Only studies published in peer-reviewed journals were considered. According to the authors’ language capabilities, English, German, Italian, French, and Spanish articles were included. Based on the Oxford Centre for Evidence-Based Medicine, only clinical trials with level I evidence were eligible. Only studies that clearly stated the molecular weight of the HA used for the injections were included. Studies that compared HA with other biologically active non-HA treatments (e.g., platelet-rich plasma, corticosteroids, mesenchymal stem cells) were also included to increase data pooling. Only studies conducted on the knee for OA were eligible. Studies that evaluated intra-articular HA injections augmented with other biologically active compounds were not considered. Studies regarded as comparators of other non-injection therapies were also not eligible.

### 2.2. Search Strategy

This study was conducted according to the Preferred Reporting Items for Systematic Reviews and Meta-Analyses: the 2020 PRISMA statement [32]. The PICOTD algorithm was preliminarily established as follows:

P (Problem): knee OA;

I (Intervention): intra-articular HA injections;

C (Comparison): UHMW-, HMW-, MMW-, LMW- HA;

(Outcomes): PROMs.

T (Timing): two weeks to six months;

D (Design): randomised controlled trial.

In November 2024, the following databases were accessed: PubMed, Web of Science, and Embase. No time constraint was set for the search. The last update was in March 2024. The Medical Subject Headings (MeSHs) used for the database search are reported in the Appendix. No additional filters were used in the database search.

### 2.3. Selection and Data Collection

Two authors (C.H.N. and F.M.) independently performed the database search. All resulting titles were screened by hand, and the abstracts were accessed if suitable. The full text of the abstracts that matched the topic was accessed. If the full text was not accessible or available, the article was not considered for inclusion. A cross-reference of the bibliography of the full-text articles was also performed for inclusion. Disagreements were debated and mutually solved by the authors. In case of further disputes, a third senior author (N.M.) took the final decision.

### 2.4. Data Items

Two authors (M.B., J.K.) independently performed data extraction. The following data at baseline were extracted: author, year of publication, journal, length of follow-up, number of patients with related mean age, and BMI. Data concerning the VAS were collected at baseline and last follow-up. Data were extracted in Microsoft Office Excel version 16.72 (Microsoft Corporation, Redmond, WA, USA). A placebo was considered to be any intra-articular injection performed with isotonic saline solution or anaesthetic. A control was considered to be any intra-articular injection performed with any biologically active compound (PRP, CCs, MSCs). Following previous reports [24,25,26], the HAs were categorised as follows: LMW (500–1500 kDa), MMW (1500–3000 kDa), HMW (3000 to 6000 kDa), and UHMW (from 6000 kDa).

### 2.5. Methodological Quality Assessment and Quality of the Recommendations

The risk of bias was assessed following the guidelines outlined in the Cochrane Handbook for Systematic Reviews of Interventions [33]. Two reviewers (G.P. and M.P.) independently evaluated the risk of bias in the included studies. Any disagreements were resolved by a third senior author (N.M.). RCTs were assessed using the revised Risk of Bias (RoB2) tool [34,35] from the Cochrane Risk of Bias framework for randomised trials. The following potential biases were evaluated: bias arising from the randomisation process, bias due to deviations from the intended interventions, bias resulting from missing outcome data, bias in outcome measurement, and bias in the selection of reported results.

### 2.6. Synthesis Methods

The main author (F.M.) performed the statistical analyses following the recommendations of the Cochrane Handbook for Systematic Reviews of Interventions [36]. IBM SPSS software version 25 (International Business Machines Corporation, Armonk, NY, USA) was used for descriptive statistics. Variance analysis (ANOVA) was used, and *p*-values > 0.1 were considered satisfactory. The analyses were divided into four time points: from two weeks to one month, one month to two months, two to four months, and four to six months. STATA Software/MP, Version 14.1 (StataCorporation, College Station, TX, USA), was used for the statistical analyses. Network analyses were performed through the STATA routine for Bayesian hierarchical random-effects model analysis. The Log Odd Ratio (LOR) effect measure was adopted to analyse dichotomic data. The overall inconsistency was evaluated through the equation for global linearity via the Wald test. If P_Wald_ > 0.5, the null hypothesis cannot be rejected, and the consistency assumption could be accepted at the overall level of each treatment. Both confidence (CI) and percentile (PrI) intervals were set at 95%. The control group was considered an active group, and the placebo was considered a comparator for the analyses. Edge plots were drawn to assess the contribution of each intervention, statistical weights, and direct comparisons. Interval plots were drawn to rank the interventions according to their effect size. A funnel plot was also drawn to assess the publication bias. Greater pyramidal symmetry in the funnel plot is associated with lower data heterogeneity.

## 3. Results

### 3.1. Subsection

**Figure 1:**
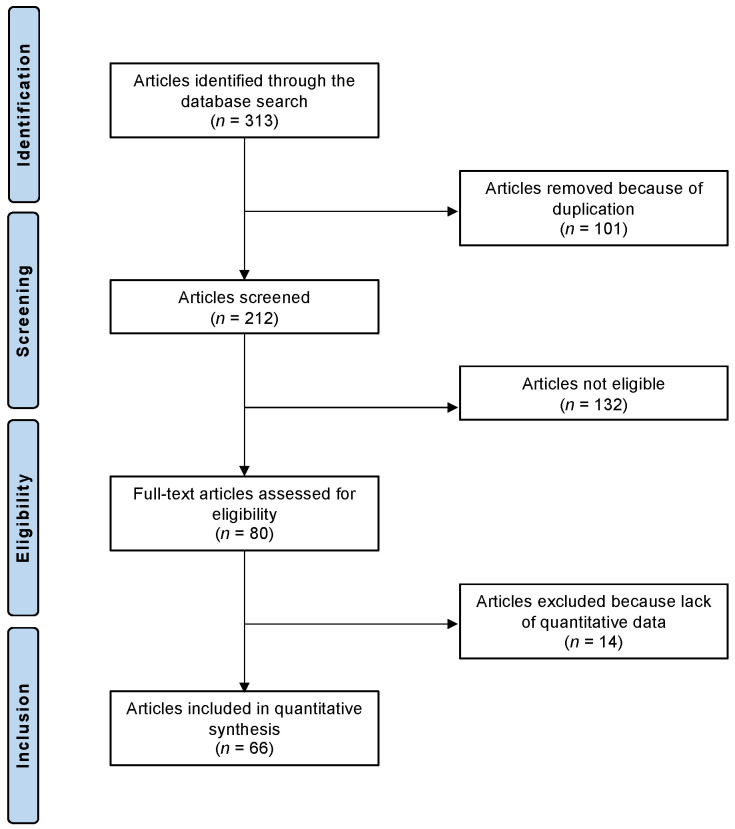
PRISMA flow chart of the literature search.

A systematic literature search identified a total of 313 clinical trials that addressed the topic of interest. Of them, 101 studies were identified as duplicates and excluded. The abstracts of the remaining 212 investigations were screened for eligibility. An additional 132 studies were not eligible. In detail, the reasons for exclusion were inappropriate study type and design (*N* = 48), low level of evidence (*N* = 21), not clearly stating the molecular weight of the HA for injection (*N* = 13), evaluating intra-articular HA injections augmented with other biologically active compounds (*N* = 19), considering other non-infiltrative therapies as comparators (*N* = 16), missing implementation of the VAS (*N* = 10), and language limitations (*N* = 5). In total, 14 more studies did not include quantitative data on the endpoints of interest and were therefore not considered. This left 66 RCTs for final inclusion. The results of the literature search are shown in Figure 1.

### 3.2. Methodological Quality Assessment

**Figure 2:**
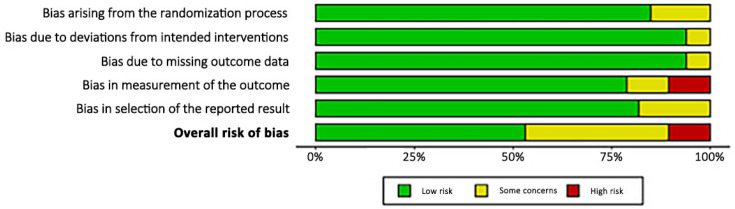
Cochrane risk of bias tool (RoB2).

The revised Cochrane risk of bias assessment tool (RoB2) was applied to investigate the risk of bias in all investigations included in the present review, as they were RCTs. The assessment identified some concerns during the randomisation process. However, given the established comparability of the groups studied at baseline, bias arising from the randomisation process was rated as predominantly low-risk. Risk of bias based on the deviations from the intended intervention, missing outcome data, the selection of the reported outcome, and the measurement of the outcome were occasionally noted with some concerns, leading to a low to moderate overall risk of bias in these domains. Given the lack of investigators blinding, some of the articles found a high risk of bias in outcome measurement; in all other studies, a low to medium risk was found for this area. In conclusion, the risk of bias graph evidenced a predominately good quality of the methodological assessment of the RCTs (Figure 2). 

### 3.3. Study Characteristics and Results of Individual Studies

Data from 9822 patients were collected. A total of 67% (6567 of 9002) of patients were women. The mean age of the patients was 62.1 ± 5.0 years, and the mean BMI was 27.8 ± 2.2 kg/m^2^. Generalities and demographics of the included studies are shown in Table 1.

#### 3.3.1. Baseline of the Groups

No difference was found in the mean age, women (%), mean BMI, or mean VAS, attesting the comparability of the data at baseline (Table 2).

#### 3.3.2. Two Weeks to One Month

**Figure 3:**
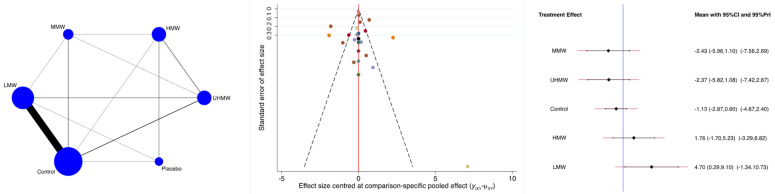
Results of the network meta-analyses (LMW: low molecular weight; MMW: medium molecular weight; HMW: high molecular weight; UHMW: ultra-high molecular weight).

The MMW and UHMW groups were associated with greater VAS reduction, while the LMW group was associated with a lower reduction (Figure 3). The P_Wald_ indicated no evidence of statistically significant inconsistency in the analyses (P_Wald_ = 0.2).

#### 3.3.3. One Month to Two Months

**Figure 4:**
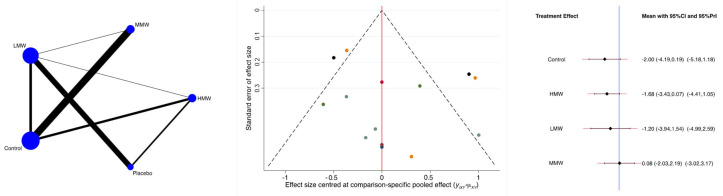
Results of the network meta-analyses (LMW: low molecular weight; MMW: medium molecular weight; HMW: high molecular weight).

The MMW HA was associated with the lowest reduction in the VAS. The other groups had similar effect sizes (Figure 4). At this time, no data for the UHMW were available. The P_Wald_ indicated no evidence of statistically significant inconsistency in the analyses (P_Wald_ = 0.3).

#### 3.3.4. Two to Four Months

**Figure 5:**
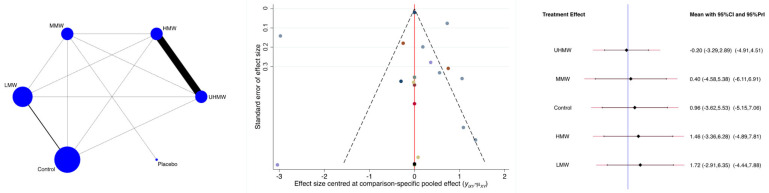
Results of the network meta-analyses (LMW: low molecular weight; MMW: medium molecular weight; HMW: high molecular weight; UHMW: ultra-high molecular weight).

The UHMW and MMW HA groups were associated with the greatest reduction in the VAS, while the LMW group was associated with the lowest reduction (Figure 5). The P_Wald_ indicated no evidence of statistically significant inconsistency in the analyses (P_Wald_ = 0.2).

#### 3.3.5. Four to Six Months

**Figure 6:**
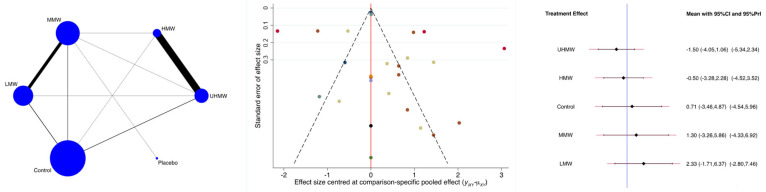
Results of the network meta-analyses (LMW: low molecular weight; MMW: medium molecular weight; HMW: high molecular weight; UHMW: ultra-high molecular weight).

The UHMW and HMW HA groups were associated with the greatest reduction in the VAS, while the LMW group was associated with the lowest reduction (Figure 6). The P_Wald_ indicated no evidence of statistically significant inconsistency in the analyses (P_Wald_ = 0.4).

## 4. Discussion

According to the main findings of the present Bayesian network meta-analysis, the current level I evidence suggests that UHMW HA injections in knee OA patients lead to the greatest reduction in the VAS, followed by HMW HA. MMW and LMW HA injections were the interventions associated with the lowest decrease in the VAS. Conservative management of knee OA remains a major challenge for every clinician, as the destructive effect on cartilage caused by OA cannot be fully reversed. The present study compared the effects of different intra-articular molecular weight HA injections at various times.

In the final comparison, which spanned four to six months, the reduction in the VAS followed the molecular weight, ranging from the greatest reduction in the UHMW group to the LMW group. After six months, the MMW and LMW HA seem to not have any beneficial effects compared to the control group, while UHMW and HMW are beneficial. Our results echo those of the network meta-analysis by Hummer et al. [13] in that higher-molecular-weight HA seem to have a beneficial effect on reported pain in patients with knee OA in short-term outcomes under six months. The decisive difference in our study is the precise distinction between different molecular weight groups. We identified definite differences regarding low, medium, high, and ultra-high MW, further distinguishing the different preparations of HA currently available and their effects. 

The American Academy of Orthopaedic Surgeons (AAOS) does not recommend intra-articular HA injections in their 2022 guidelines for the non-operative management of knee osteoarthritis (third edition) or their previous 2013 version [98,99]. In the 2022 guidelines, no subset of patients who benefit from intra-articular HA could be identified, and different MWs or compositions of HA were not taken into account [99]. HMW HA seems to show a short-term beneficial effect in another network meta-analysis with combined injections of intra-articular agents, such as the combination of HMW HA and platelet-rich plasma, especially in the first six months [12]. In the previous network analysis, all intra-articular agents performed better than saline except for ozone. Both meta-analyses describe different results over time depending on the molecular weight but predominantly positive results in the HMW/UHMW groups. Those varying results regarding the MW groups at various intervals could be explained by the MW’s biological effects on HA. HMW and UHMW HA closely resemble the HA found within the joint, considering that HA slowly diminishes as OA progresses [100]. LMW/MMW HA showed stronger immunostimulatory activities in vitro cell models while absorbing faster than HMW or UHMW HA [101]. Therefore, this results in relatively better short-term results, as shown around the two weeks to one-month mark and worse results in the mid-term outcome around the six-month mark. Other systematic research highlights the short-term beneficial effects of HA injections regardless of the MW and or preparation [102]. 

In summary, the intra-articular administration of HA seems beneficial in patients with knee OA, depending on its molecular weight. The risks associated with intra-articular HA injections are mild, including allergic reactions, pain, infection, and, rarely, numbness, headaches, and dizziness [103]. The present Bayesian network meta-analysis followed the standard protocol for assessing level I randomised control trials. The inclusion criteria included the application of the revised Cochrane risk of bias assessment tool (RoB2), which showed a predominantly good quality of the methodological assessment regarding the included RCTs. Furthermore, comparability at baseline was good, as supported by the results of the ANOVA test. No statistically significant inconsistency in the P_Wald_ analyses could be found in all comparisons. 

Limitations to note are the short follow-up period of 6 months and the lack of studies on long-term comparisons of different intra-articular MW HA injections. Only four RCTs reported a follow-up period over 12 months [44,45,46,88]. Differences in the injection protocol present a bias risk: single injections, staged injections with different latency times between injections, and combined injections were included. Thus, these issues represent important limitations regarding a definitive statement on single injection or the use of only two or more stages of HA. Furthermore, differences in the threshold definition of molecular weight can blur the boundaries between the MW groups, especially around the LWM to MMW groups, as these thresholds are close together and overlap between different studies. Previous interventions are rarely reported and can influence the reported results. Only the VAS pain score was analysed in the present study, and it did not include the functional status or patient scores. This results from the fact that most studies only reported data on pain, not on functional outcomes. Moreover, the absence of functional and QoL outcomes as a limitation acknowledges the importance of these outcomes for a complete understanding of treatment effectiveness and suggests directions for future research. Depending on the treatment type and the acid composition, two categories can be distinguished: linear and cross-linked HA. The cross-link is a process that allows linear HA molecules to be linked to obtain new structures consisting of several HA filaments with a higher molecular weight. With the cross-linking technique, molecular weights of 6 MDalton (average HA molecular weight of a healthy joint) can be reached up to real gels, in which the individual molecules have lost their individuality. However, given the lack of quantitative data, cross-linked HA was not considered for analysis. Given the lack of quantitative data, it was not possible to analyse additional follow-ups. As pointed out by Hermans et al. [104], most studies included populations over 60, while a benefit for the working-age population could be shown.

Most of the included RCTs lacked detailed information regarding the severity of OA, and subgroup analyses were not conducted. The present Bayesian network meta-analysis focuses primarily on pain, measured using the VAS, rather than radiographic assessments of OA severity. In addition, the focus on pain reduction does not fully capture the broader effects of intra-articular HA injections. Functional status, quality of life, and patient satisfaction are essential outcomes that would provide a more comprehensive evaluation of treatment efficacy. Future studies should include these additional outcomes to offer a more thorough assessment of HA injections and their impact on patient’s daily functioning and well-being. Future research should prioritise consistently reporting patient-reported outcomes to elucidate the relationship between disease stage and treatment efficacy. While the present Bayesian network meta-analysis provides insights into the more effective molecular weights of HA injections, the lack of consistency in injection protocols across trials remains a limitation that warrants further investigation. Some RCTs used a single intra-articular HA injection, while others employed multiple injections over a variable timeframe for similar molecular weights. The diversity in injection protocols introduces heterogeneity in the results, complicating data interpretation and impacting generalizability. Additionally, the timing and number of injections could influence the onset and duration of symptom relief, further contributing to variability. An international consensus and recommendations on the most appropriate protocol are necessary. Future studies should standardise injection protocols, comparing single and multiple injections and assessing optimal regimens. Another potential limitation of this study is the inclusion of studies with a high risk of bias, as assessed using the Cochrane RoB2 tool. While these studies were not excluded, they may have influenced the results. In future work, a sensitivity analysis should be conducted to evaluate the impact of excluding high-risk studies on the robustness of the conclusions. More high-level evidence needs to emerge on this topic to improve the evidence on intra-articular injections with HA.

## 5. Conclusions

In the presented Bayesian network meta-analysis, UHMW HA was the intervention associated with the greatest reduction in the VAS, followed by HMW six months after intervention. MMW and LMW HA fell short of the control group at six months post intervention.

## Figures and Tables

**Table 1:** Generalities and demographics of the included studies (FU: follow-up; UHMW: ultra-high molecular weight; HMW: high molecular weight; MMW: medium molecular weight; LMW: low molecular weight).

Author, Year	Journal	Last FU (Months)	Intervention	Patients (*n*)	Mean Age (*y*)	Women (%)	Mean BMI (kg/m^2^)
Altman et al., 2004 [37]	*Osteoarthr. Cartil.*	6	UHMW	173	62.9	46	W 29.8; M 29.0
			Placebo	174	63.3	64	
Altman et al., 2009 [38]	*Semin. Arthritis Rheum.*	6	HMW	293	62.5	63	32.4
			Placebo	295	60.8	63	33.0
Arden et al., 2014 [39]	*Curr. Med. Res. Opin.*	2	UHMW	108	64.5	55	W 28.2; M 26.4
			Placebo	110	60.9	46	W 28.1; M 26.9
Arliani et al., 2021 [40]	*Rev. Bras. Ortop. (Sao Paulo)*	6	Control	14	62.8	79	28.3
			HMW	15	63.4	87	28.1
Bahrami et al., 2020 [41]	*BMC Musculoskelet. Disord.*	6	MMW	44	56.0	72	27.1
			LMW	46	59.5	75	27.5
Berenbaum et al., 2012 [25]	*Ann. Rheum Dis.*	6	LMW	217	67.2	14	28.0
			LMW	209	66.1	13	27.7
Bongkotphet et al., 2009 [42]	*J. Health Res.*	3	MMW	32	64.8	69	26.1
			HMW	32	64.8	69	26.1
Buendía-López et al., 2018 [43]	*J. Orthop. Traumatol.*	12	Control	35	56.2	52	24.9
			UHMW	36	56.6	53	24.9
			Control	35	57.4	52	25.2
Cerza et al., 2012 [44]	*Am. J. Sports Med.*	24	Control	60	66.5	58	
			LMW	60	66.2	53	
Chen et al., 2021[45]	*Stem Cell Res. Ther.*	96	HMW	8	70.5	63	25.5
			Control	17	67.7	82	27.7
			Control	17	68.6	88	26.7
			Control	15	64.9	80	25.7
Cole et al., 2017 [46]	*Am. J. Sports Med.*	52	Control	52	55.9	43	27.4
			HMW	59	56.8	60	29.0
Cubukçu et al., 2005 [47]	*Clin. Rheumatol.*	8	HMW	20	52.6	70	
			Placebo	10	57.6	100	
DeCaria et al., 2011 [48]	*Arch. Gerontol. Geriatr.*	6	LMW	15	71.9	47	30.5
			Placebo	15	72.9	47	29.4
Diraçoğlu et al., 2009 [49]	*J. Back Musculoskelet. Rehabil.*	1	HMW	42	59.4	90	31.1
			Placebo	21	56.2	100	31.3
Diraçoğlu et al., 2016 [50]	*J. Back Musculoskelet. Rehabil.*	6	MMW	21	58.0	80	30.5
			LMW	20	56.4	85	30.8
Dougados et al., 1993 [51]	*Osteoarthr. Cartil.*	12	LMW	55	67.0	78	
			Placebo	55	69.0	65	
Dulic et al., 2021 [52]	*Medicina (Kaunas)*	12	Control	123	56.9	49	28.6
			HMW	35	59.4	57	30.0
			Control	37	58.8	29	28.5
Duymus et al., 2017 [53]	*Knee Surg. Sports Traumatol. Arthrosc.*	12	Control	41	60.4	97	27.6
			MMW	40	60.3	97	28.4
			Control	39	59.4	89	27.6
Galluccio et al., 2021 [21]	*Ther. Adv. Musculoskelet. Dis.*	6	LMW	30	66.0	47	
			LMW	30	64.0	47	
			LMW	30	64.0	57	
Guler et al., 2014 [54]	*Eur. J. Orthop. Surg. Traumatol.*	6	MMW	86	55.1	89	28.6
			Control	89	55.0	80	28.4
Guo et al., 2018 [20]	*Med. Sci. Monit.*	6	UHMW	129	64.8	77	27.4
			HMW	129	62.0	73	27.6
Ha et al., 2017 [55]	*BMC Musculoskelet. Disord.*	3	MMW	141	62.4	81	24.8
			UHWM	146	62.0	78	25.1
Hangody et al., 2018 [19]	*Cartilage*	6	LMW	149	57.5	65	28.9
			MMW	150	59.2	66	28.4
			Placebo	69	58.0	74	29.1
Henderson et al., 1994 [56]	*Ann. Rheum Dis.*	1	LMW	10	63.9	50	
			LMW	25	72.1	80	
			Placebo	20	60.0	75	
			Placebo	26	67.0	69	
Ho et al., 2022 [57]	*J. Orthop. Translat.*	12	Control	10	56.7	60	25.4
			HMW	10	59.1	80	26.0
Huang et al., 2011[58]	*BMC Musculoskelet. Disord.*	6	LMW	100	65.9	74	25.7
			Placebo	100	64.2	78	25.4
Huang et al., 2021 [59]	*BMC Musculoskelet. Disord.*	12	UHMW	71	56.6	65	
			LMW	71	56.0	71	
Huskisson et al., 1999 [60]	*Rheumatology (Oxford)*	6	LMW	50	65.8	76	
			Placebo	50	64.8	58	
Juni et al., 2007 [61]	*Arthritis Rheum*	6	HMW	222	63.3	65	28.2
			MMW	219	63.5	69	28.1
			MMW	219	63.3	65	28.6
Karlsson et al., 2002 [62]	*Rheumatology (Oxford)*	12	LMW	92	72.0	67	
			HMW	88	70.0	65	
			Placebo	66	71.0	61	
Ke et al., 2021[63]	*BMC Musculoskelet. Disord.*	6	Placebo	220	61.6	78	25.4
			HMW	218	61.5	77	25.6
Khanasuk et al., 2012 [64]	*J. Med. Assoc. Thai.*	6	HMW	16	65.1	80	26.6
			LMW	16	67.0	80	25.4
Kim et al., 2023 [65]	*Sci. Rep.*	4	Control	30	63.6	83	
			MMW	30	65.4	75	
Ko et al., 2022 [66]	*Pharmaceutics*	12	UHMW	71	66.1	79	
			UHMW	71	65.5	82	
Lin et al., 2019 [67]	*Arthroscopy*	12	Control	31	61.2	71	24.0
			MMW	27	62.5	66	26.6
			Placebo	29	62.2	63	25.0
Louis et al., 2018 [68]	*Arthroscopy*	6	Control	24	53.2	42	25.6
			UHMW	24	48.5	54	27.0
Maheu et al., 2019 [69]	*PLoS ONE*	6	MMW	144	67.1	72	26.4
		6	HMW	148	66.6	61	26.3
Maia et al., 2019 [23]	*Clinics (Sao Paulo)*	6	MMW	16	56.6	63	31.9
			Control	12	60.3	92	31.4
Martin Martin et al., 2016 [70]	*BMC Musculoskelet. Disord.*	6	Control	32	69.4	86	27.2
			LMW	32	70.0	65	27.3
Mochizuki et al., 2020 [71]	*Asia Pac. J. Sports Med. Arthrosc. Rehabil. Technol.*	2	LMW	37	69.0	68	23.1
			MMW	36	65.2	71	24.5
Moon et al., 2023 [72]	*Pain Med.*	4	Control	30	67.5	63	
			MMW	30	67.5	70	
			UHMW	30	67.0	93	
Ozcamdalli et al., 2017 [73]	*Cartilage*	6	HMW	10			
			Control	10			
Park et al., 2021 [74]	*Am. J. Sports Med.*	6	Control	55	60.6	71	25.5
			UHMW	55	62.3	85	25.9
Paterson et al., 2016 [75]	*BMC Musculoskelet. Disord.*	3	Control	12	49.9	27	27.9
			HMW	11	52.7	30	30.9
Petrella et al., 2002 [76]	*Arch. Intern. Med.*	3	LMW	30	67.3	36	29.5
			Control	30	66.3	42	29.4
			Placebo	30	62.6	43	32.7
Petrella et al., 2015 [77]	*BMC Musculoskelet. Disord.*	6	Control	33	59.0	63	29.8
			HMW	32	59.0	50	29.0
Petterson et al., 2019 [78]	*Knee Surg. Sports Traumatol. Arthrosc.*	6	MMW	184	59.5	59	29.9
			Placebo	185	58.7	57	30.4
Pham et al., 2004 [79]	*Ann. Rheum Dis.*	12	MMW	131	64.9	71	
			Control	85	64.5	69	
			Placebo	85	64.9	61	
Raeissadat et al., 2015 [80]	*Clin. Med. Insights Arthritis Musculoskelet. Disord.*	12	Control	87	56.9	10	28.2
			LMW	73	61.1	24	27.0
Raeissadat et al., 2017 [81]	*Clin. Med. Insights Arthritis Musculoskelet. Disord.*	6	Control	41	57.0	82	28.6
			LMW	36	59.5	82	27.5
Raeissadat et al., 2018 [82]	*J. Pain Res.*	6	Control	87	58.1	75	26.8
			LMW	87	61.1	76	28.6
Raeissadat et al., 2020 [83]	*J. Pain Res.*	12	Control	60	57.1	72	27.9
			LMW	59	58.6	71	28.7
Raeissadat et al., 2021 [84]	*BMC Musculoskelet. Disord.*	12	Control	59	56.1	73	27.5
			Control	60	57.9	76	27.5
			LMW	59	56.1	75	27.4
			Control	60	57.6	75	27.0
Sanchez et al., 2012 [85]	*Arthroscopy*	6	Control	89	60.5	52	27.9
			HMW	87	58.9	52	28.2
Sconza et al., 2023 [86]	*Int. J. Mol. Sci.*	6	Control	26	68.0	42	29.1
			LMW	26	68.0	62	27.9
Shimizu et al., 2010 [87]	*J. Orthop. Sci.*	6	LMW	32	75.9	78	
			Control	29	75.3	73	
Su et al., 2018 [88]	*Clin. Rheumatol.*	18	Control	28	50.7	63	28.2
			Control	26	54.2	56	28.2
			LMW	32	53.1	60	28.7
Sun et al., 2017 [89]	*J. Bone Jt. Surg. Am.*	6	UHMW	66	62.7	77	24.7
			HMW	66	62.5	71	25.2
Tammachote et al., 2016 [90]	*J. Bone Jt. Surg. Am.*	6	HMW	55	62.6	86	26.3
			Control	55	61.0	73	25.8
Tasciotaoglu et al., 2003 [91]	*Clin. Rheumatol.*	6	MMW	30	57.4		32.7
			Control	30	60.1		33.3
Vanelli et al., 2010 [92]	*Knee Surg. Sports Traumatol. Arthrosc*	3	Control	30	60.0	66	26.7
			LMW	30	67.0	67	28.8
Waluyo et al., 2021 [93]	*J Rehabil Med.*	3	Control	44	62.6	77	
			LMW	32	62.0	71	
Wang et al., 2018 [94]	*Exp. Ther. Med.*	6	Control	60	63.6	77	25.3
			LMW	60	62.5	73	26.0
Wang et al., 2022 [95]	*Medicina (Kaunas)*	6	Control	58	61.9	78	24.1
			UHMW	58	63.0	71	24.0
van der Weegen et al., 2014 [96]	*J. Arthroplast.*	6	MMW	99	58.7	51	28.6
			Placebo	97	60.1	48	29.3
Yaradilmis et al., 2020 [97]	*J. Orthop.*	12	MMW	35	63.0	87	32.4
			Control	36	60.3	87	31.3
			Control	34	58.9	90	32.5

**Table 2:** ANOVA test to assess baseline comparability.

Endpoints	Sum of Square	Mean Square	F Statistic	*p*-Value
Age	100.74	33.58	1.43	0.24
Women	0.14	0.05	1.77	0.16
BMI	39.13	13.04	2.47	0.07
VAS	3605.01	1201.67	1.94	0.14

## Data Availability

The datasets generated during and/or analysed during the current study are available throughout the manuscript.

## Abbreviations

Osteoarthritis (OA), hyaluronic acid (HA), randomised controlled trials (RCTs), visual analogue scale (VAS), ultra-high molecular weight (UHMW), high molecular weight (HMW), medium molecular weight (MMW), low molecular weight (LMW), risk of bias (RoB), analysis of variance (ANOVA), Log Odd Ratio (LOR), confidence interval (CI), percentile interval (PrI).
